# Opportunities to encourage mail order pharmacy delivery service use for diabetes prescriptions: a qualitative study

**DOI:** 10.1186/s12913-019-4250-7

**Published:** 2019-06-25

**Authors:** Julie A. Schmittdiel, Cassondra J. Marshall, Deanne Wiley, Christopher V. Chau, Connie M. Trinacty, J. Frank Wharam, O. Kenrik Duru, Andrew J. Karter, Susan D. Brown

**Affiliations:** 1 Division of Research, Kaiser Permanente Northern California, 2000 Broadway, Oakland, CA 94612 USA; 2 University of California, Berkeley, CA USA; 3 Center for Health Research, Kaiser Permanente Hawaii, Honolulu, HI USA; 4 Harvard Medical School and Harvard Pilgrim Health Care Institute, Boston, MA USA; 5 University of California, Los Angeles, CA USA

**Keywords:** Diabetes, Mail order pharmacy, Barriers and facilitators, Patient preference, Acceptance of health care, Qualitative research

## Abstract

**Background:**

Medication non-adherence is a major contributor to poor outcomes in diabetes. Previous research has shown an association between use of mail order pharmacy delivery and better medication adherence, but little is known about the barriers and facilitators to mail order pharmacy use in diabetes patients. This qualitative study examined factors related to mail order pharmacy use versus traditional “brick and mortar” pharmacies to refill prescriptions.

**Methods:**

We conducted four 90-min focus groups in 2016 among 28 diabetes patients in the Hawaii and Northern California regions of Kaiser Permanente, a large integrated health care delivery system. We queried participants on their preferred mode for refilling prescriptions and perceived barriers and facilitators of mail order pharmacy use. One researcher independently coded each focus group transcript, with two of these transcripts double-coded by a second researcher to promote reliability. We employed thematic analysis guided by the Capability, Opportunity, Motivation, and Behavior (COM-B) framework using NVivo 11 software.

**Results:**

A total of 28 diabetes patients participated. Participants’ average age was 64.1 years; 57% were female; and racial/ethnic backgrounds included Asian/Native Hawaiian/Pacific Islander (36%), Black/African-American (21%) Hispanic/Latino (7%), and non-Hispanic White (36%). Analysis uncovered 26 themes related to the decision to use mail order pharmacy, with each theme representing a barrier or facilitator mapped to the COM-B framework. Most themes (20/26) fell into the COM-B category of ‘Opportunity.’ Opportunity barriers to mail order pharmacy use included unpredictability of medication delivery date, concerns about mail security, and difficulty coordinating refill orders for multiple prescriptions. In contrast, facilitators included greater access and convenience (e.g., no need to wait in line or arrange transportation) compared to traditional pharmacies. Motivational facilitators to mail order pharmacy use included receiving a pharmacy benefit plan incentive of a free one-month supply of prescriptions.

**Conclusions:**

This study found that while patients with diabetes may benefit from mail order pharmacy use, they perceive numerous barriers to using the service. These findings will inform the design of interventions and quality improvement initiatives to increase mail order pharmacy use, which in turn may improve medication adherence and outcomes in diabetes patients, across health care systems.

**Electronic supplementary material:**

The online version of this article (10.1186/s12913-019-4250-7) contains supplementary material, which is available to authorized users.

## Background

Over 30 million Americans have type 2 diabetes [1], a condition associated with significant mortality and morbidity. Diabetes is currently the seventh leading cause of death in the United States [2], and the number of adults with the disease has gone up dramatically in the past two decades [3].

Good medication adherence is associated with improved health outcomes, fewer hospitalizations, and lower mortality in patients with diabetes [4–6]. However, only 50–70% of patients with chronic conditions are adherent to their regular medications [7–9]. Numerous studies have shown significant disparities in medication adherence among diabetes patients; minorities and low socioeconomic status (SES) patients with diabetes have poorer adherence to cardiometabolic medications shown to reduce diabetes complications [10–15].

Use of mail order pharmacy services to deliver medications, or mail order pharmacy use, accounts for at least an estimated 25% of pharmacy sales in the US, and rates are even higher among Americans ages 65 and up [16]. Growth in mail order pharmacy services for outpatient prescription drugs has increased significantly since the 1980s; prior to this time, community-based pharmacies only occasionally offered ad hoc home delivery of medications to small, selected groups of patients based on factors such as patient home address and mobility [16]. A growing body of research suggests that mail order pharmacy use is correlated with better medication adherence in diabetes [17–21] and across a wide range of chronic conditions [21–26]. Mail order pharmacy use is also associated with better health care outcomes and decreased health care utilization and costs [18, 23, 27–29]. Patients who use mail order pharmacy are more likely to be non-Hispanic White and have higher SES [17, 28]. Encouraging mail order pharmacy use in diverse populations may be an important tool for reducing disparities in health care outcomes [25].

Despite the evidence that mail order pharmacy may be an important strategy for improving diabetes care and outcomes, there is little to no published evidence identifying factors that influence patients’ uptake of this service. This qualitative study sought to identify and describe facilitators and barriers to mail order pharmacy use among diverse diabetes patients across two health systems. The purpose of this study was to inform patient outreach strategies for a pragmatic clinical trial designed to increase mail order pharmacy use among diabetes patients (clinical trial.gov registration number NCT02621476).

## Methods

### Study setting

This study is set within two health care delivery systems that represent significant geographic, demographic, and structural diversity. Kaiser Permanente Northern California (KPNC) is a non-profit, integrated, group-model health care delivery system that in 2016 served close to 4 million members in a 13-county area of Northern California. This population is more than 30% non-white, 20% have attained a high school or lower level of education, and almost 50% have a household income <$50 K annually. Kaiser Permanente Hawaii (KPHI) is a non-profit, mixed model health maintenance organization that in 2016 served nearly 230,000 members throughout Hawaii. This population is 70% non-white, representing one of the most ethnically and racially diverse populations in the United States; 16% have attained a high school or lower level of education, and about 45% have a household income <$50 K annually. The patient membership of both health systems is representative of the demographic characteristics of the geographic areas the health systems serve.

### Mail order pharmacy use at Kaiser Permanente (KP)

KPNC and KPHI have over 120 local “brick and mortar” pharmacies, located on-site within outpatient clinics and hospital facilities. KPNC and KPHI both maintain KP-run mail-order pharmacy distribution systems in coordination with the local KP pharmacies. Although most new prescriptions are filled in the local KP pharmacy, KP patients can elect to receive their prescription refills sent via mail order pharmacy, ordered either through the online patient portal (kp.org) or a toll-free pharmacy refill telephone service. KP patients have telephone access to a pharmacist to answer any medication-related questions one-on-one regardless of whether a refill was dispensed via mail order pharmacy or a local KP pharmacy. There is no minimum days’ supply required for mail order delivery. KP typically dispenses 90–100 day medication supplies for most oral diabetes prescriptions through both mail order and local KP pharmacies. Some patients have a financial incentive to use mail order pharmacy through their health insurance pharmacy benefit structure. This is typically receiving 3 copayments’ worth of medication for the price of 2 copayments (e.g., 3 months’ supply for the price of 2 months’ supply) when the medications are refilled by mail.

### Study participants

Potential participants were identified using KPNC and KPHI electronic health record data. Patients were considered eligible if they had one primary inpatient diagnosis or 2 outpatient diagnoses of type 2 diabetes (ICD9 codes 250.xx, 357.2, 362.0x, 366.41), were adults ages 18–89, and had filled an oral diabetes medication prescription (alpha-glucosidase inhibitor, amylin analog, biguanide, glucagon-like peptide-1 (GLP-1) receptor agonist, meglitinide, sodium-glucose cotransporter-2 (SGL2) inhibitor, sulfonylurea, thiazolidinedione (TZD)) between 7/1/2014 and 6/30/2015. Insulin users who had filled one of these oral diabetes medication prescriptions were included; insulin-only users were excluded. Since the study was conducted in English, non-English speakers were excluded. To identify influences on the use of both mail order and local pharmacies, we sought the perspectives of both current mail order pharmacy users and non-users. We defined mail order pharmacy users as those who had used the mail order pharmacy at least once during the 12-month period prior to recruitment using KP prescription refill databases; the 12-month window was used to reduce recall bias when patients conveyed their experiences with the service.

### Study design and implementation

Patients were recruited via mailed letter and telephone outreach and offered $50 as an incentive for participation. We invited patients to participate in one of four 90-min focus groups in February and March of 2016, in either Oakland, CA (KPNC) or Honolulu, HI (KPHI). We used a 2 (site: KPNC vs. KPHI) × 2 (mail order pharmacy user vs. non-user) design to examine patient perspectives across health systems and across levels of familiarity with mail order pharmacy. We selected focus group methodology to elicit a broad range of unique ideas (rather than promote consensus) and to identify concepts that may inform future interventions promoting uptake of mail order pharmacy services. Participants provided written informed consent.

The research team included professionals from health services, public health, epidemiology, medicine, behavioral science, pharmacy, clinical administration, and project management backgrounds. This purposefully interdisciplinary team fostered our ability to identify a range of potential influences on mail order pharmacy use. Focus groups were conducted by three trained facilitators, one in KPNC and two in KPHI. The researchers, including interviewers, were unknown to research participants prior to data collection. To improve neutrality and consistency of responses across groups [30], we used standardized semi-structured interview guides using the same predetermined open-ended questions and moderator prompts [31]. Interview questions queried participants about their pharmacy use preferences and the perceived benefits and disadvantages of both mail order and local pharmacies (please see the Additional file 1). Focus groups were digitally recorded, professionally transcribed verbatim, and deidentified prior to analysis. To augment qualitative data and describe the sample, we extracted demographic and clinical data from electronic health records. The study was approved by the KPNC Institutional Review Board; KPHI ceded Institutional Review Board (IRB) oversight for this study to KPNC. A completed ‘Standards for Reporting Qualitative Research’ checklist is included as supplemental material for reference.

### Qualitative data analysis

We employed thematic analysis [32] to inductively and deductively derive themes from qualitative data [33] using NVivo 11 software (QSR Intl Pty Ltd.; Doncaster, Australia). We used the “Capability, Opportunity, Motivation, and Behavior” model (COM-B) [34] and closely related, validated Theoretical Domains Framework (TDF) [35, 36] to guide our qualitative analyses. These frameworks synthesize leading theories of behavior change, providing a systematic method for identifying influences on a given behavior. One doctoral candidate and two doctoral-level researchers in the fields of public health and behavioral medicine reviewed all transcripts to develop a coding scheme of broad meaningful themes. Guided by the COM-B framework, we focused on themes relevant to patients’ barriers and facilitators enacting two target behaviors: using mail order pharmacy and using a local pharmacy. We further defined each target behavior as encompassing three individual actions: requesting a prescription refill (e.g., in-person, by phone, or online), paying for a prescription refill (e.g., using a credit card online), and receiving a prescription refill (e.g., refilling it in-person from a pharmacist or receiving it in the mail). One researcher independently coded each focus group transcript, with two of these transcripts double-coded by a second researcher to promote reliability. Following the coding process, themes were mapped to COM-B categories [34]: psychological capability (e.g., knowledge of the mail order pharmacy service); physical opportunity (e.g., the pharmacy location); social opportunity (e.g., the opportunity to interact with a pharmacist); automatic motivation (e.g., emotional reactions, rewards); and reflective motivation (e.g., conscious beliefs and intentions). The research team discussed the text allocated to each theme, and the themes allocated to each category, with discrepancies resolved by consensus.

## Results

Table 1 shows the demographic characteristics of study participants (*n* = 28). Slightly more than half were female (57%); the average age of participants was 64.1 years. Thirty-six percent of patients were Asian, Native Hawaiian or other Pacific Islander; 21% were Black/African-American; 7% were Hispanic/Latino, and 36% were non-Hispanic White.

**Table 1:** Demographic characteristics of focus group attendees overall at Kaiser Permanente Northern California (KPNC) and Kaiser Permanente Hawaii (KPHI) (*n* = 28)

Demographics	n (%)
Female	16 (57%)
Age (mean, sd, range)	64.1, 10.0, 37–83
Race/Ethnicity	
Asian/Native Hawaiian/Pacific Islander	10 (36%)
Black/African-American	6 (21%)
Non-Hispanic White	10 (36%)
Hispanic/Latino	2 (7%)
Mail Order Users	19 (68%)
Site	
KPNC	16 (57%)
KPHI	12 (43%)

**Fig. 1:**
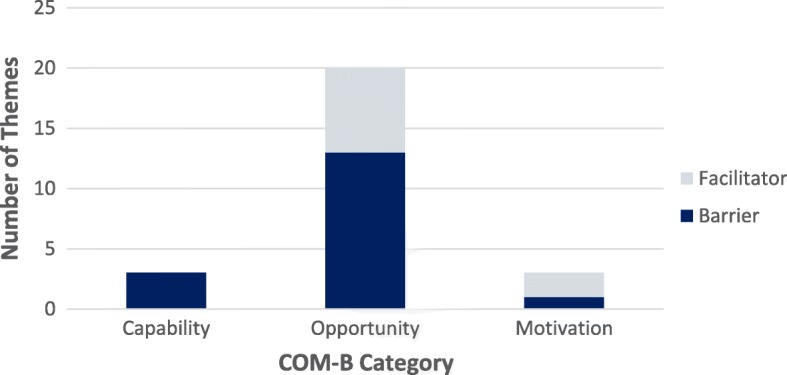
Number of Focus Group Themes by COM-B Category

Table 2 shows the 26 qualitative themes that emerged from the analysis, mapped to COM-B categories and stratified by perceived barriers vs. facilitators of using mail order pharmacy. As shown in Fig. 1, which displays the broad grouping of themes by COM-B category, most themes (20/26) fell into the category of ‘Opportunity.’ Of those, 13 themes (65%) represented barriers. These ‘opportunity’ barriers included unpredictability of the medication delivery date, concerns about mail security, and difficulty coordinating refill orders for multiple prescriptions. Patients’ quotes related to this theme appear in italics; the focus group type (mail order user versus (vs.) non-user), site, and participant number from which they emerge follow in parentheses:

*You don’t know when you going to get it. They cannot give you a delivery date. […] I can’t do it, because I’m too scared about that, […] it cost me too much, too expensive to take that chance with insulin [of failing to receive it on time]. (Non-user) KPNC, 6.*

*So there’s a little apprehension on my part, because right now I currently live in a business district [where] the mail gets tampered with. (Non-user) KPHI, 2.*

*And then, if your mailbox is not in a safe place – because there’s been mail theft – you could lose your drugs through mail theft. And if you paid for the drug, I don’t know that they’re going to, you know, replenish it even though it was stolen and even though you paid for it. So those are the drawbacks. (User) KPHI, 6.*

*I have eight prescriptions, and because of the change of the medicines here and there, they’ve gotten all out of sync. So, I’m ordering eight different times in a three-month period, and that means I have to process eight different claims with the insurance company to be reimbursed for them. (User) KPNC, 1.*

**Table 2:** Barriers and Facilitators to Refilling Medications Using Mail Order Pharmacy: Focus Group Themes Mapped to the Capability, Motivation, Opportunity Model of Behavior Change (COM-B)

	Barriers	Facilitators
Capability	Theme (#Focus groups; type)^a^	Theme (#Focus groups; type)^a^
Psychological	• Lack of knowledge about how mail order process works (4; *user* and *non-user*) • Not planning ahead (3; *user* and *non-user*) • Limited technological literacy (2; *user* and *non-user*)	
Opportunity		
Physical	• Mail order system (e.g., online or phone) is unreliable, inconsistent, or hard to navigate (2; *user*) • Longer wait times when refill requires provider authorization (1; *user*) • Wanting to use different forms of payment, but mail order system requires one credit card to stay on file (1; *non-user*) • Unpredictable delivery date (1; *non-user*) • Difficult to coordinate refill dates for multiple prescriptions (1; *user*) • Need for certain technology (e.g., computer) to use mail order system (2; *user* and *non-user*) • Concerns about mail security (e.g., possible theft) (4; *user* and *non-user*) • Possibility of receiving refill ‘too late’ (i.e., after medication has run out) because patients must time their order to match the mail order system’s allowable window to order (1; *user*) • Required to go to pharmacy if refill requires provider authorization (1; *user*) • Mail order system cannot accommodate special requests (e.g., early refill due to upcoming travel) (1; *user*) • Inability to get refills immediately (i.e., same day) (1; *user*) • Not all medications can be refilled through the mail order system (2; *user*)	• Mail order system is reliable, consistent, easy to navigate (2; *user*) • Arrives quickly in the mail (1; *user*) • No lines or waiting in-person at pharmacy (2; *user*) • No travel required to obtain refill (2; *user*) • Reminders to pick up refill from post office (1; *user*) • Notification from health system that refill is on the way (1; *user*)
Social	• No availability of in-person consultation with pharmacist (1; *non-user*)	• Option to avoid negative interpersonal interactions (e.g., with pharmacy staff) (1; *user*)
Motivation		
Automatic		• One-month supply of prescription refill is free when using mail order (3; *user* and *non-user*)
Reflective	• Belief that prescription may be negatively impacted (e.g., spoil, ‘go bad’) if left outside upon delivery (2; *user* and *non-user*)	• Confidence in ability to use mail order system (2; *user*)

^a^Number of focus groups in which the theme emerged; type of focus group (mail order pharmacy *user* and/or *non-user*)

In contrast, ‘opportunity’ facilitators included greater access and convenience (e.g., no need to wait in line or arrange transportation) compared to traditional pharmacies, and finding the mail order pharmacy system easy to use. For example:

*What’s good is that, once you’ve put in all your information, you can just sort of hit the, you know, the basic control [to automate the process]. So, like, your credit card, you don’t have to put in numbers each time. (User) KPHI, 2.*

*Kaiser’s website is very well organized and put together, and it’s a breeze, really. (User) KPHI, 8.*

Social opportunity factors—specifically, the ability to avoid interactions with pharmacy staff— emerged as a facilitator of mail order pharmacy use:

*Which is why I would rather use the mail-order service, because then you don’t have to deal with these overworked clerks. (User) KPHI, 7.*

Others described positive interactions with pharmacy staff, and therefore viewed lack of interaction as a barrier to using mail order pharmacy:

*I have gone into the pharmacy, and they’ve, like, just recently, they gave me a prescription…and I look at it when I get it – it was different. Okay? Now, this particular time, I was able to ask about it, and they said, “Oh, they changed manufacturers, and it’s the same medication.” But a couple of years ago, they had actually given me the wrong one. And if that happened with a mail order, and it’s something that I needed, you couldn’t, like, right then fix it. (Non-user) KPNC,5.*

Within the ‘motivation’ category, barriers to using mail order pharmacy included worry that prescriptions, including diabetes-specific medications such as insulin, would be negatively impacted (e.g., “go bad”) if left outside upon delivery:

*So I was kind of concerned about that, if it sat in the mailbox too long. Is it really effective, or is it ineffective? (User) KPHI, 10.*

*Also, I don’t know how good it would be for insulin to be shipped, because I don’t know how long it might sit in my mailbox before I get to it…. because it’s supposed to be kept cool…. And if it’s sitting in this tin mailbox in the beating sun…* – (*Non-user*) KPNC, 5.

In contrast, facilitators included confidence in one’s ability to use the mail order pharmacy ordering system and receiving an incentive of a free one-month supply of medication each time a 3-month prescription was ordered:

*Well, previously, when you had the mail order, I did participate. And, at that time, they offered, if you ordered 90 days, you’d get 30 days free. I liked that benefit. (Non-user) KPHI,2.*

Further probing for the above patient confirmed that they had used mail order pharmacy more than 12 months ago but were non-users during the 12 month look back window, and that repeated address and PO box changes had discouraged them from using the service more recently.

*…if they just going to approximate the way you’re going to save money from parking or a bus or whatever, things like that, it does – for me, it’s not enough marketing to convince me….But if it’s real savings, like it’s a lower cost to me, then I might look at it. (Non-user) KPNC, 16.*

*“I think the best reason that nobody has mentioned [within the focus group] yet is that you get the third month free. And it’s a tremendous savings.” (User)KPHI,2.*

*I started mail order for the free month. And when I started, I was taking only two medications. Now I take six, so if you listed how much savings, that, you know, it would be in the hundreds of dollars that getting that extra, you know, month for each medication. You know, because I’m a type 2 diabetic. I’m a heart patient. You know, so I take six medications a day. So the savings for me is tremendous, you know? That’s why I do mail order. And now, online, it’s so easy, you know? It’s so easy that I can’t see another way to do it, you know, because it would cost, you know, like I said, a couple hundred dollars at least. (User) KPHI, 12.*

Finally, within the ‘capability’ category, barriers included lack of knowledge about how mail order pharmacy works (e.g., the processes of ordering and delivery) and lack of technological literacy:

*If you need that certain medication that you have to go down and sign for it, how are you going to get that through the mail? [...] there’s some that they say is like a [controlled] substance, like, pain and stuff like that. To refill that, you have to go down to the pharmacy to get it signed. How would you be able to get that through the mail? The same, you know, for refills. (Non-user) KPHI, 5.*

*I didn’t even know that you [could] call and [order] over the phone…(User) KPHI, 2.*

*Not everybody is computer literacy, you know. So, like, at my house, we have a computer, but I will say, “Hon, can you do this for me? Hon, can you do that for me?” But it’s where – if I go on a computer and I look for prescriptions, I wouldn’t know which one to press. (Non-user) KPHI, 8*.

## Discussion

Mail order pharmacy use is associated with better medication adherence and health care outcomes in diabetes patients, but little is known about patients’ barriers and facilitators to mail order use. Our focus groups and qualitative analysis showed that some diabetes patients may encounter important barriers to leveraging the opportunity to use mail order pharmacy, including difficulties using the ordering system, order accuracy, concerns about mail box security, longer **waiting** times for receiving medication compared to in-person refills, and concerns that insulin delivery via mail would compromise the safety and efficacy of the medication. One survey study of patients between the ages of 65 and 79 found that seniors’ concerns about mail order pharmacy use included the potential for lost or stolen medications, receiving the correct medication, and concerns that outdoor exposure may harm the prescription medication’s efficacy [16]. Our study uncovered similar concerns, and contributes to the evidence based with the new additional finding that these concerns may apply to diabetes-specific medications such as insulin. Another study that interviewed veterans living with AIDS/HIV found patients were concerned their medications might run out before a new prescription fill arrived in the mail; this finding was echoed in our analysis as well. Still, prior studies suggest that mail order pharmacy users are more satisfied with pharmacy services than traditional community ‘brick and mortar’ pharmacy users [37–39]; addressing the barriers to mail order pharmacy use thus has the potential to increase satisfaction while improving health care outcomes and reducing costs. Health care systems and pharmacy benefit managers should address these issues to improve the patient experience and encourage mail order pharmacy use in those patients who could benefit from the service.

Patients in our study cited numerous facilitators that encouraged opportunities to use the mail order pharmacy, including not having to wait in line in-person or having to travel to the pharmacy to refill their medications. Patients whose benefits included a mail order pharmacy incentive to receive an extra copayment’s worth of medication day’s supply for free when paying for two copayment’s worth (e.g. three for the price of two) and using mail order saw this as a facilitator as well. Since 90–100 day fills of diabetes medication are standard in these settings, most diabetes patients eligible for this incentive could likely benefit. This finding is consistent with that of a prior focus group study focused on understanding drug benefit decisions among adults ages 65 and older, which found that access to mail order pharmacy services was a valued attribute in prescription drug plans [40]. Promoting these attributes that can encourage patients to consistently use mail order pharmacy services, and expanding the drug benefit that allows for greater days’ supply for similar cost when using mail order, may be approaches to increase the uptake of prescription home delivery [41].

It is important to note that patients had a variety of perspectives on mail order pharmacy and themes occasionally contradicted one another. For example, some patients felt the mail order pharmacy system was unreliable and the delivery date unpredictable, whereas others felt the system was reliable and cited delivery notifications as facilitating its use. In another example, the need for in-person interactions with pharmacists and staff was cited as both a barrier and facilitator to mail order pharmacy use. Patients’ experience and understanding of mail order pharmacy services will not be universal, and health care systems should target outreach and communication to those who may be more in need of information. In addition, mail order pharmacy services should continue to offer the opportunity for one-on-one consultations with pharmacists to address medication questions and concerns, and allow patients to fill prescriptions at brick-and-mortar pharmacies in person if that is their personal preference [15].

This study has limitations that should be noted. The focus group format and structure did not allow for understanding differences in results by patient characteristics such as age, gender, or socioeconomic status; prior studies suggest preference for mail order pharmacy vs. community pharmacy may vary by some of these characteristics [17, 42]. However, a strength of our study was the diversity of the participants who participated in the focus groups; a majority were non-Hispanic White. Social desirability bias in pharmacy services research [43] may have led patients to underemphasize the barriers to mail order pharmacy use within their health care system. However, the number of barriers uncovered as well as the consistency with prior research lends validity to these results. This study defined non-users of mail order pharmacy as those who had not used mail order for refills within the past 12 months; it is possible that “non-users” could have had experience with mail order pharmacy in the more distant past.

This study focused on diabetes patients; patients with other conditions may have different experiences with mail order pharmacy. However, other studies of mail order pharmacy use in patients with different chronic conditions have found similar themes. This study took place in two integrated delivery systems within the United States. Results might not be fully generalizable to other health care delivery systems, and the organizational roles and responsibilities for improving mail order pharmacy services may be different in other settings, particularly those that are less integrated or that don’t operate their own pharmacy services. Finally, we are unable to compare the effectiveness of the mail order pharmacy services examined in this study to those in other settings or countries, as these data are unavailable.

## Conclusions

Our study found that while patients with diabetes may benefit from mail order pharmacy use, they perceive numerous barriers and facilitators to using the service. These findings derived through this qualitative work, can be used to design interventions and quality improvement initiatives to increase mail order pharmacy use and promote improved medication adherence and outcomes in diabetes patients, across health care systems.

## Additional file

Additional file 1: Focus Group Interview Questions. Questions for Mail Order and Non-Mail Order Pharmacy Users (DOC 37 kb)

## Data Availability

The qualitative datasets analysed during the current study are not publicly available since consent for sharing data was not granted by participants; de-identified data may be available from the corresponding author on reasonable request.

## Abbreviations

COM-B: Capability, opportunity, motivation, and behavior
GLP-1: Glucagon-like peptide-1
IRB: Institutional review board
KP: Kaiser Permanente
KPHI: Kaiser Permanente Hawaii
KPNC: Kaiser Permanente Northern California
SES: Socioeconomic status
SGL2 inhibitor: Sodium-glucose cotransporter-2
TDF: Theoretical domains framework
TZD: Thiazolidinedione
