# Supplementary material for: Opportunities to encourage mail order pharmacy delivery service use for diabetes prescriptions: a qualitative study
Source: BMC Health Serv Res. 2019 Jun 25;19:422. doi: 10.1186/s12913-019-4250-7 (PMC6593516; doi:10.1186/s12913-019-4250-7)
Supplement: Supplementary file 1 — Focus Group Interview Questions. Questions for Mail Order and Non-Mail Order Pharmacy Users (DOC 37 kb) [file 12913_2019_4250_MOESM1_ESM.doc]

**Focus Group Interview Questions**

**Focus Group Questions for Mail Order Pharmacy Users**

1. Can you talk about the last time you used the mail order pharmacy to refill prescriptions? How did that work out for you?
2. What are some of the reasons that you use the mail order pharmacy to refill your prescriptions?
3. How did using mail order pharmacy work out for you?
4. What specific aspects of using mail order pharmacy made things easier for you?
5. How was using mail order pharmacy difficult for you?
6. What specific aspects of using mail order pharmacy make things difficult for you?
7. What about mail order pharmacy do you prefer compared to using your local Kaiser pharmacy?
8. What aspects of your local Kaiser pharmacy do you prefer versus compared to mail order pharmacy?
9. Do any of you use both mail order pharmacy and the local Kaiser pharmacy?
10. Can you tell me about the reasons for using both?
11. How would you encourage someone to use mail order pharmacy services like you do?
12. Overall, on a scale from 1 to 10—with 1 being “totally unacceptable” and 10 being “perfectly acceptable”—how acceptable would a message be that contains personal information from your medical record?
13. Any additional comments?

**Focus Group Interview Questions for Non-Mail Order Pharmacy Users**

1. Most of us do not always refill our prescriptions on time, and as a result, we may sometimes run out of prescription for a few days. Please think back to an instance when you did not refill your prescription on time. What were some of the reasons that you did not refill your prescription on time?
2. Can you talk about your most recent experience refilling a prescription at a local Kaiser pharmacy?
3. How did using your local Kaiser pharmacy benefit you?
4. What specific aspects of using your local Kaiser pharmacy made things easier for you?
5. What difficulties did you encounter in refillng a prescription at your local Kaiser pharmacy?
6. What specific aspects of using your local Kaiser pharmacy made things more difficult for you?
7. Have you ever used the mail order pharmacy to refill medications?
8. If yes, what was your experience?
9. Thinking back to your last few visits to your local KP pharmacy to pick up a prescription refill, can you think of a how using mail order pharmacy might benefit you?
10. What specific aspects of using mail order pharmacy would make things easier for you?
11. Remembering those same last few visits to a local KP pharmacy, can you think of how using mail order pharmacy might be more difficult than going to the pharmacy?
12. Are there specific reasons you would not use mail order pharmacy to refill your prescription?
13. If you believe mail order pharmacy is not for you, how could it be changed in a way that would make you more willing to use it?
14. What information would help you decide whether to use mail order pharmacy services to refill prescriptions?
15. Overall, on a scale from 1 to 10—with 1 being “totally unacceptable” and 10 being “perfectly acceptable”—how acceptable would a tailored message be that contains personal information from your medical record?
16. Any additional comments?
